# Comparison of post-transplantation diabetes mellitus incidence and risk factors between kidney and liver transplantation patients

**DOI:** 10.1371/journal.pone.0226873

**Published:** 2020-01-10

**Authors:** Vidit N. Munshi, Soroush Saghafian, Curtiss B. Cook, K. Tuesday Werner, Harini A. Chakkera

**Affiliations:** 1 PhD Program in Health Policy, Harvard University, Cambridge, Massachusetts, United States of America; 2 Harvard Kennedy School, Harvard University, Cambridge, Massachusetts, United States of America; 3 Mayo Clinic Arizona, Scottsdale, Arizona, United States of America; Universidad de Navarra, SPAIN

## Abstract

**Background:**

Most prior studies characterizing post-transplantation diabetes mellitus (PTDM) have been limited to single-cohort, single-organ studies. This retrospective study determined PTDM across organs by comparing incidence and risk factors among 346 liver and 407 kidney transplant recipients from a single center.

**Methods:**

Univariate and multivariate regression-based analyses were conducted to determine association of various risk factors and PTDM in the two cohorts, as well as differences in glucometrics and insulin use across time points.

**Results:**

There was a higher incidence of PTDM among liver versus kidney transplant recipients (30% vs. 19%) at 1-year post-transplant. Liver transplant recipients demonstrated a 337% higher odds association to PTDM (OR 3.37, 95% CI (1.38–8.25), p<0.01). 1-month FBG was higher in kidney patients (135 mg/dL vs 104 mg/dL; p < .01), while 1-month insulin use was higher in liver patients (61% vs 27%, p < .01). Age, BMI, insulin use, and inpatient FBG were also significantly associated with differential PTDM risk.

**Conclusions:**

Kidney and liver transplant patients have different PTDM risk profiles, both in terms of absolute PTDM risk as well as time course of risk. Management of this population should better reflect risk heterogeneity to short-term need for insulin therapy and potentially long-term outcomes.

## Introduction

Health care providers face an important tradeoff in managing patients who have undergone solid organ transplantation. Corticosteroids and immunosuppressive agents such as tacrolimus and sirolimus prevent graft rejection. However, these medications bring significantly increased risk for development of impaired glucose tolerance and post-transplantation diabetes mellitus (PTDM), also known as new onset diabetes after transplant (NODAT). PTDM is a potentially serious complication of solid organ transplantation affecting patients without a prior history of a diabetes diagnosis, leading to decreased graft function and survival, increased risk of cardiovascular disease, and increased healthcare costs [1, 2].

Most clinical recommendations on PTDM emphasize the need for prevention of organ rejection while maintaining standards of care for those who develop concurrent PTDM [3, 4]. However, the current literature leaves significant gaps in our understanding of PTDM and the potential improvements we can make in its management. Recommendations on management of PTDM [1, 5] do not vary by the type of solid organ transplanted even though evidence suggests that the characteristics and outcomes of PTDM across organ sites are inconsistent. For instance, incidence estimates of PTDM vary widely, ranging from 4–25% in kidney transplantations, 2.5–25% in liver transplantations, and 4–40% in heart transplantations [4, 6–9]. These wide ranges in estimates make it difficult to understand the true burden of PTDM, which can potentially impact the 30,000 solid organ transplantations that take place in the United States each year [10, 11]. The discrepancies in incidence have been attributed to changing definitions of PTDM and preferred treatment strategies over time, patient follow-up time, and presence of risk factors in available data sets, among others. For these reasons, it is likely that PTDM incidence, and by extension, its burden on the healthcare system, is being under-estimated [12].

In recent years, availability of transplant patient data has allowed for analyses to better understand the pre-transplant risk factors for PTDM [13–21]. However, these studies come from a wide range of hospitals and health systems, and typically focus on a single organ cohort such as kidney transplantations. However, the mechanism underlying PTDM, namely the use of immunosuppressive agents in reducing rejection risk, also plays a major role in PTDM risk across other solid organ transplantations. For institutions that are comprehensive solid organ transplant centers—ones that perform more than one type of organ transplant—it is important to know how PTDM risk may vary by organ type, and know the common and unique risk factors for PTDM, so that post-transplant care can be planned. To this end, there has been no study comparing PTDM risk by type of organ transplanted with the goal of motivating differential management in these populations.

We introduce a dataset of liver transplantation patients to complement a dataset of kidney transplantation patients previously used in published analyses by Boloori et al. [22]. Together, these datasets allow, for the first time, a comparison of data across transplant types from the same medical institution with respect to PTDM risk and factors that might affect its incidence. While similarities in PTDM features are expected due to common risk factors shared by all solid organ types (e.g. obesity and immunosuppressant use), we aim to (a) characterize and better understand any differences that may exist across these populations, and (b) identify what these differences may imply for predicting PTDM risk or need for hyperglycemia therapy. These, in turn, can shed light on strategies and potential for guideline-making that may be effective in improving this patient population’s health outcomes [23].

## Materials and methods

### Study population

This was a retrospective study of a dataset compiled through a de-identified chart review with IRB (Mayo Clinic IRB) approval (Continuing Review #: PR16-008236-03). One dataset consisted of 346 patients who underwent first time liver-only transplant between 2007 and 2012. Information collected on each patient included demographic data and medical history, as well HbA1c, fasting blood glucose (FBG), and diabetes mellitus (DM) status pre-transplant and at 1-, 4-, and 12-months post-transplant. In addition, we collected type of hypoglycemic medication administered to each patient, type of immunosuppressant used, corticosteroid use, and trough level of immunosuppressive agent at each time point post-transplant. We also utilized a previously published data set of 407 kidney transplant patients who underwent transplant between 1999 and 2006 at the same institution [24]. This data set included the nearly same information as the liver data set. In some cases, variables were included in the kidney dataset but not in the liver dataset due to differences in data collection protocol based on organ type and information gained from extracting certain lab values. For example, status of patients’ livers who are due for liver transplant alone would indicate abnormal values for cholesterol and so variables such as cholesterol and triglyceride are not typically collected.

### Summary of immunosuppression protocols

In renal transplants, the standard immunosuppression protocol is induction therapy with either rabbit anti-thymocyte, immumoglobulin, or basiliximab. All patients typically receive a 5-day tapering course of glucocorticoids. Subsequently, most receive a steroid-free maintenance immunosuppression using mycophenolate mofetil, and tacrolimus is instituted. For patients undergoing a liver transplant, an immunosuppression protocol of intravenous corticosteroids, tacrolimus, and mycophenolate mofetil is used postoperatively. Corticosteroid administration follows the regimen of 500 mg intravenous methylprednisolone preoperatively, 50 mg twice daily on postoperative day 0; and 25 mg twice daily on postoperative day 1, then progressively tapered to a treatment stop by 4 months, but may continue in certain patients with autoimmune hepatitis. Mycophenolate mofetil stops at four months unless the patient has renal impairment.

### Data validation and PTDM definition

Data were reviewed to remove significant outliers that indicated human error in data collection (e.g., lab values with a mis-placed decimal point), with only a minimal number (less than 1%) of observations removed from the dataset. In concordance with previous literature [5, 22], we defined PTDM as patients who met at least one of the following three criteria: Fasting blood glucose level > 126 mg/dL, hemoglobin (HbA1c) ≥ 6.5%, or use of insulin therapy.

### Statistical analysis

We report means and standard deviations for available variables of interest determined as those found in previous literature to be predictive of PTDM risk [22]. Differences in means were tested for statistical significance using standard t-tests. Logistic regression models were employed to describe the strength of association between these variables, as well as other treatment decisions at the 1-month follow-up, and on PTDM incidence at any point over the course of the first year after transplant. Our regression models adjust for age, sex, race, body mass index (BMI), pre-transplant HbA1c, transplant year, transplant donor status (living or deceased), and type of organ transplanted. We also describe other outcomes of interest split by organ type and by each follow-up period. These outcomes include percentage of patients classified as having PTDM (defined as having fasting blood glucose > 126 or HbA1c > 6.5) and the percentage of patients on insulin, tacrolimus, and steroids.

A substantial percentage of both renal and liver transplant patients without preexisting DM develop hyperglycemia while still hospitalized. Consequently, insulin is often required at discharge. For example, 87% of renal transplant patients included in this analysis without pre-existing DM exhibited hyperglycemia while hospitalized, and 66% were discharged on insulin [24]. Additionally, 90% of liver transplant patients from this cohort had hyperglycemia, and 59% required insulin at discharge [25]. Neither inpatient glycemic control nor insulin requirements while in the hospital have been considered in prior PTDM risk analyses. Therefore, inpatient hyperglycemia and insulin use were included as variables in the analyses. Both the patient stay mean glucose and the mean glucose 24h prior to admission were calculated as previously described [26]. Glucose data represent point-of-care (capillary measurements) performed using the Accucheck Inform (Roche Diagnostics). Insulin use in the hospital was obtained from the electronic medical record.

## Results

### Patient characteristics

Table 1 gives descriptive statistics on patients undergoing renal or liver transplant considering only the patients in the study who did not have a prior history of diabetes (n = 291 and n = 250 for kidney and liver, respectively). From this total, 55 (19%) kidney patients and 76 (30%) liver patients developed PTDM by the end of the 1-year follow-up. Univariate analysis demonstrated several differences between the kidney and liver transplant groups. Those in the kidney group were significantly younger, had fewer men, were less likely to be white, had less steroid use at 1 month, and had less insulin use at 1 month (all p < .01). In addition, renal transplant patients had lower BMI, had more live donors, had slightly but significantly higher HbA1c levels pre-transplant, and lower patient stay mean glucose levels during the hospital stay following transplant (all p < .01). The proportion of patients with liver and kidney transplant developing PTDM was significantly different (p < .01) with liver patients more likely to develop PTDM.

**Table 1:** Comparison of characteristics between renal and liver patients without pre-transplant diabetes.

Characteristic	Kidney (n = 291)	95% CI	Liver (n = 250)	95% CI	p-value
Age, y	49 (15)	(47, 51)	54 (9)	(53, 55)	< .01
Male, %	56	(51, 62)	67	(61, 73)	< .01
White, %	71	(66, 76)	88	(84, 92)	< .01
Pre-transplant BMI, kg/m^2^	26.8 (5.6)	(26.1, 27.4)	28.2 (5.6)	(27.5, 28.9)	< .01
Live donor (%)	68	(62, 73)	22	(17, 27)	< .01
Pre-transplant HbA1c, %	5.4 (0.3)^a^	(5.4, 5.5)	5.2 (0.5)^b^	(5.1, 5.3)	< .01
Steroid Use at 1 month, %	47	(42, 53)	98	(97, 100)	< .01
Insulin Use at 1 month, %	6	(3, 9)	36	(30, 42)	< .01
Inpatient mean glucose post-transplant, mg/dL	132 (19)	(129, 134)	148 (17)	(145, 150)	< .01
Mean glucose 24 hours before hospital discharge, mg/dL	136 (39)	(132, 141)	144 (28)	(140, 148)	< .01
Developed PTDM by 1 year, %	19	(14, 23)	30	(25, 36)	< .01

Reported values are mean (SD) or percentages

^a^Available for 239 patients

^b^Available for 187 patients

### Differences in timing of hyperglycemia incidence

Table 2 summarizes the eight different permutations of hyperglycemia (yes or no) that can be obtained across the three follow-up visits. For example, column 1 represents the outcome of “No-No-No” corresponding to a patient who was not hyperglycemic at any of the three follow-ups. In other words, in Column 1, 191 patients (73.8%) obtained this outcome in the kidney group compared to 103 (50%) in the liver group. Column 2 represents the “Yes-No-No” outcome corresponding to a patient who was hyperglycemic at the 1-month follow-up but not at either of the other follow-ups. Finally, column 8 represents the “Yes-Yes-Yes” outcome of hyperglycemia at all three follow-ups. Compared to kidney transplant recipients, a much lower percentage of liver transplant recipients did not meet the hyperglycemia criteria at any of the 3 follow-ups (50% in liver vs 73.8% in kidney). Conversely 10.2% of liver recipients met the criteria at *all* 3 follow-ups, compared to 8.1% of renal patients. Transient hyperglycemia, that is hyperglycemia which only occurs in the immediate post-transplant period (1-month) followed by remission, accounted for a large percentage of the difference, with 17.5% of liver patients meeting the criteria only in month 1 compared to 6.2% in the kidney group. However, if transient hyperglycemia is not included in the analysis as suggested by the 2014 International Consensus Meeting [5], the percentage of patients who experience hyperglycemia during at least 1 follow-up is still higher in the liver population (31.5% in liver compared to 20% in kidney).

**Table 2:** Number and percentage of liver and kidney transplantation patients satisfying criteria for all combinations of hyperglycemia outcomes at each follow-up.

	Satisfying the criteria for hyperglycemia							
Time	1	2	3	4	5	6	7	8
**Month 1**	No	Yes	No	No	Yes	Yes	No	Yes
**Month 4**	No	No	Yes	No	Yes	No	Yes	Yes
**Month 12**	No	No	No	Yes	No	Yes	Yes	Yes
**# of patients (%)**								
**Kidney**	191 (73.8)	16 (6.2)	8 (3.1)	5 (1.9)	9 (3.5)	6 (2.3)	3 (1.2)	21 (8.1)
**Liver**	103 (50)	36 (17.5)	5 (2.4)	9 (4.4)	20 (9.7)	5 (2.4)	7 (3.4)	21 (10.2)

### Patterns and comparisons of glycemic measures

**Fig 1:**
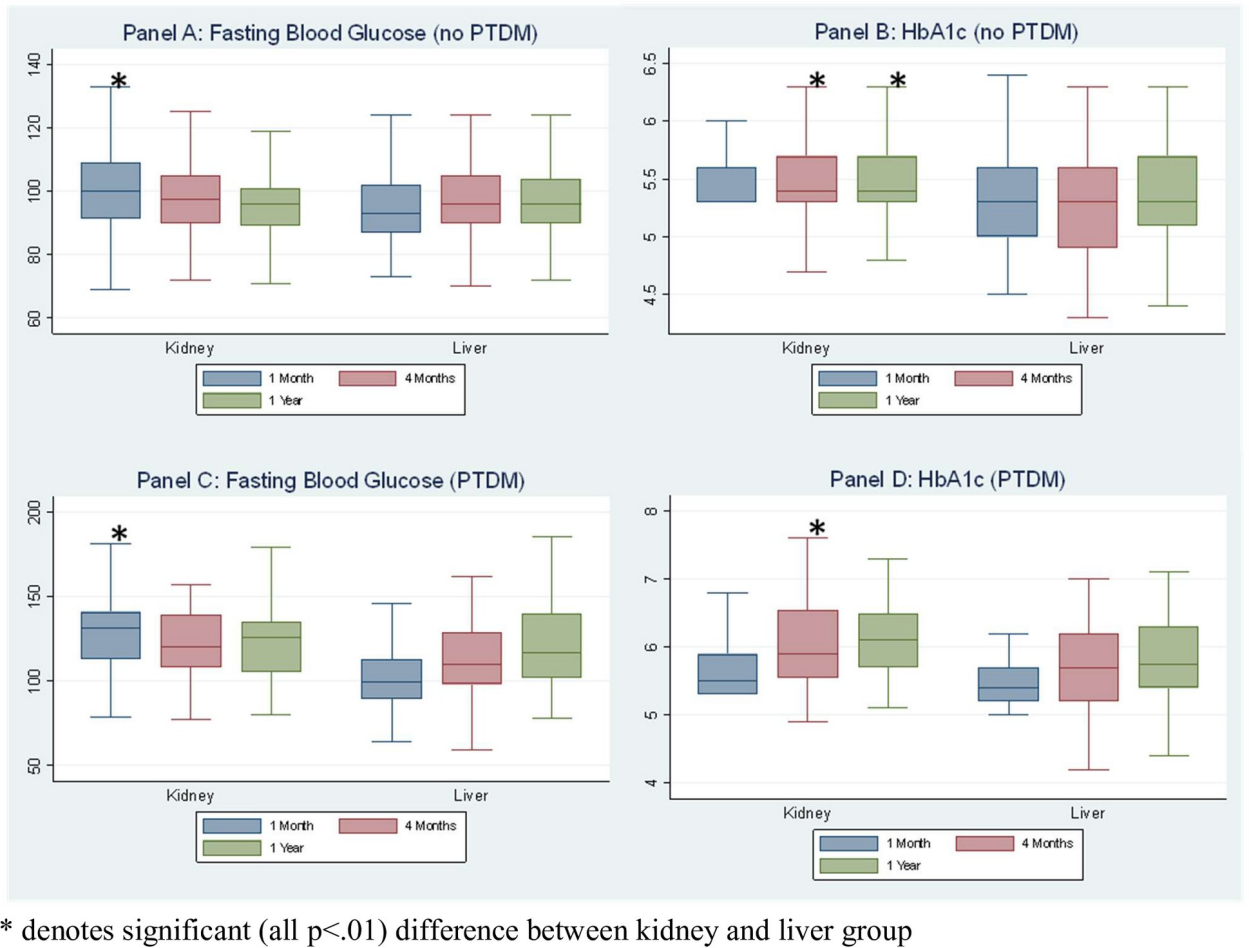
Average fasting blood glucose (Panel A) and HbA1c (Panel B) at 1 month, 4 month, and 1 year follow-ups by transplant type for patients without PTDM; Average fasting blood glucose (Panel C) and HbA1c (Panel D) at 1 month, 4 month, and 1 year follow-ups by transplant type for patients with PTDM.

Fasting glucose and HbA1c values for patients who did not develop PTDM and those who did are shown in Fig 1. Among patients who did not develop PTDM, fasting blood glucose levels were significantly higher in kidney patients compared to liver patients at 1month (p < .01), while no difference was detected at 4 and 12 months. Mean glucose in the kidney transplant group declined over the 1 year period (P < .01), while in the liver transplant patients, no significant change was found over time. HbA1c (Fig 1B) in the non-PTDM cohort were unchanged across the follow-up periods. Mean (SD) HbA1c levels at the 4 month and 12 month follow up periods were significantly lower among liver transplant patients compared to kidney transplants: 5.3% (.05) vs 5.5% (.04) at 4 months (p < .01), and 5.4% (.05) vs 5.5% (.03) at 12 months (p < .01). Mean HbA1c was not significantly different at the 1 month follow up (p = .09).

Fasting blood glucose values of patients who did develop PTDM increased over the follow-up period in the liver group, but did not change significantly in the kidney transplant cohort (Fig 1C). The 1 month, 4 month, and 12 month mean (SD) glucose values in the liver patients were 104 mg/dL (20.32), 117 mg/dL (32.57), and 125 mg/dL (35.92), respectively (p < .01). The average glucose values for the kidney cohort at the same time points were 136 mg/dL (39.55), 143 mg/dL (19.3), and 124 mg/dL (25.60; p = .07). Glucose values were significantly higher among kidney vs. liver transplant patients at 1-month only [135 mg/dL vs 104 mg/dL; p < .01]. HbA1c in the PTDM population increased significantly over the 3 time points in both the kidney (p = .01) and liver cohorts (p = .02). Mean HbA1c values were higher in the kidney cohort at 4 months (6.1 vs 5.7, p < .01), but no significant difference was detected at 1 or 12 months (Fig 1D).

### Patterns of insulin therapy

**Fig 2:**
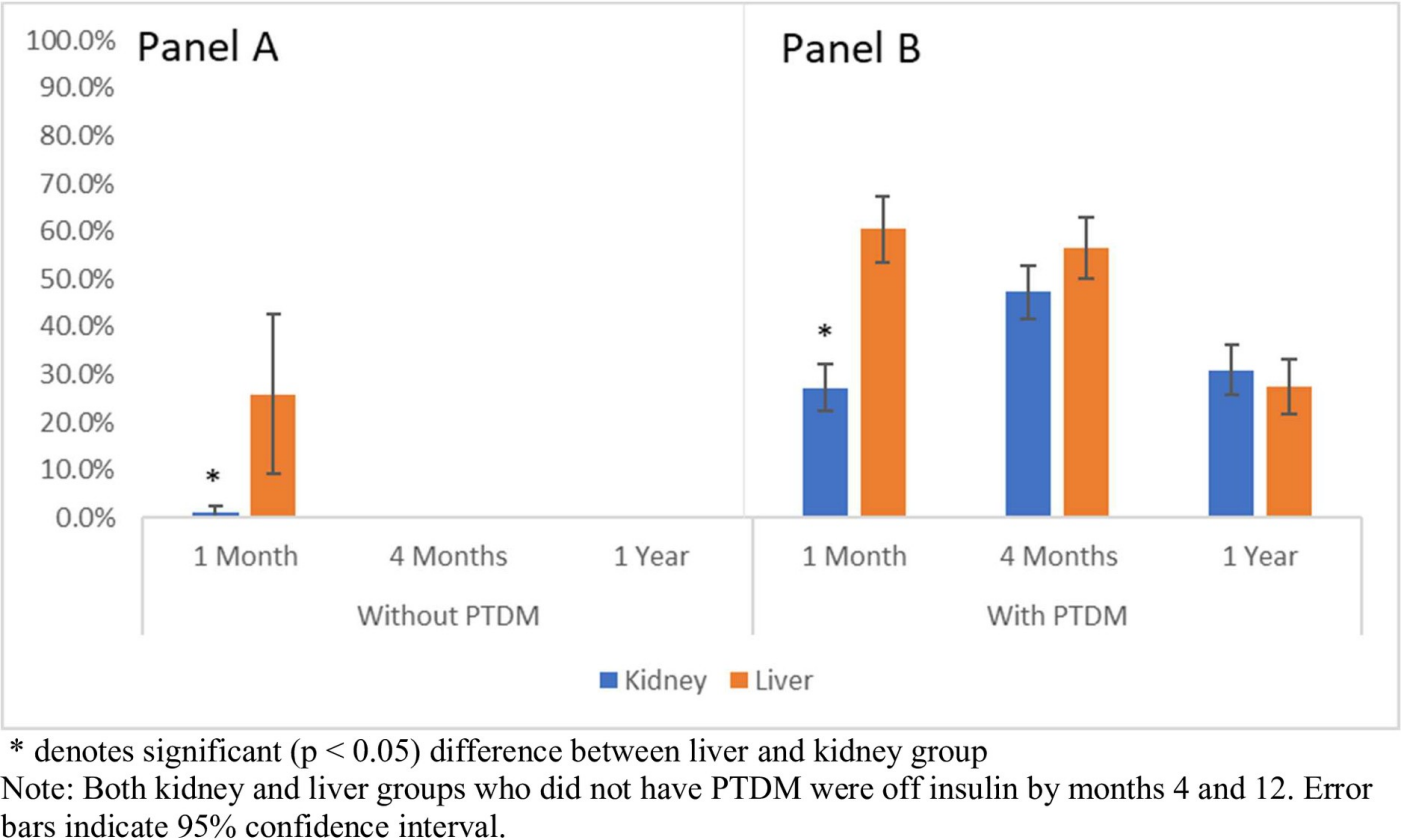
Percentage of transplant patients without (Panel A) and with (Panel B) PTDM taking insulin at 1, 4, and 12-month follow-ups.

Among patients who did not develop PTDM (Fig 2A), more liver transplant patients were on insulin compared to the kidney transplant cohort at the 1 month follow-up, (26% vs 1%, p < .01). Both groups were off insulin by months 4 and 12. In patients who developed PTDM (Fig 2B), insulin use was also higher among liver recipients compared to kidney patients (61% vs 27%, p < .01) at 1 month only. The percentage of patients on insulin in the liver group decreased with 61% at 1 month, 57% at 4 months, and 28% at 12 months (p < .01) on insulin. In the kidney group, insulin use again remained relatively constant with 27% at 1 month, 47% at 4 months, and 31% at 12 months (p = .57).

### Patterns of immunosuppressive therapy

**Fig 3:**
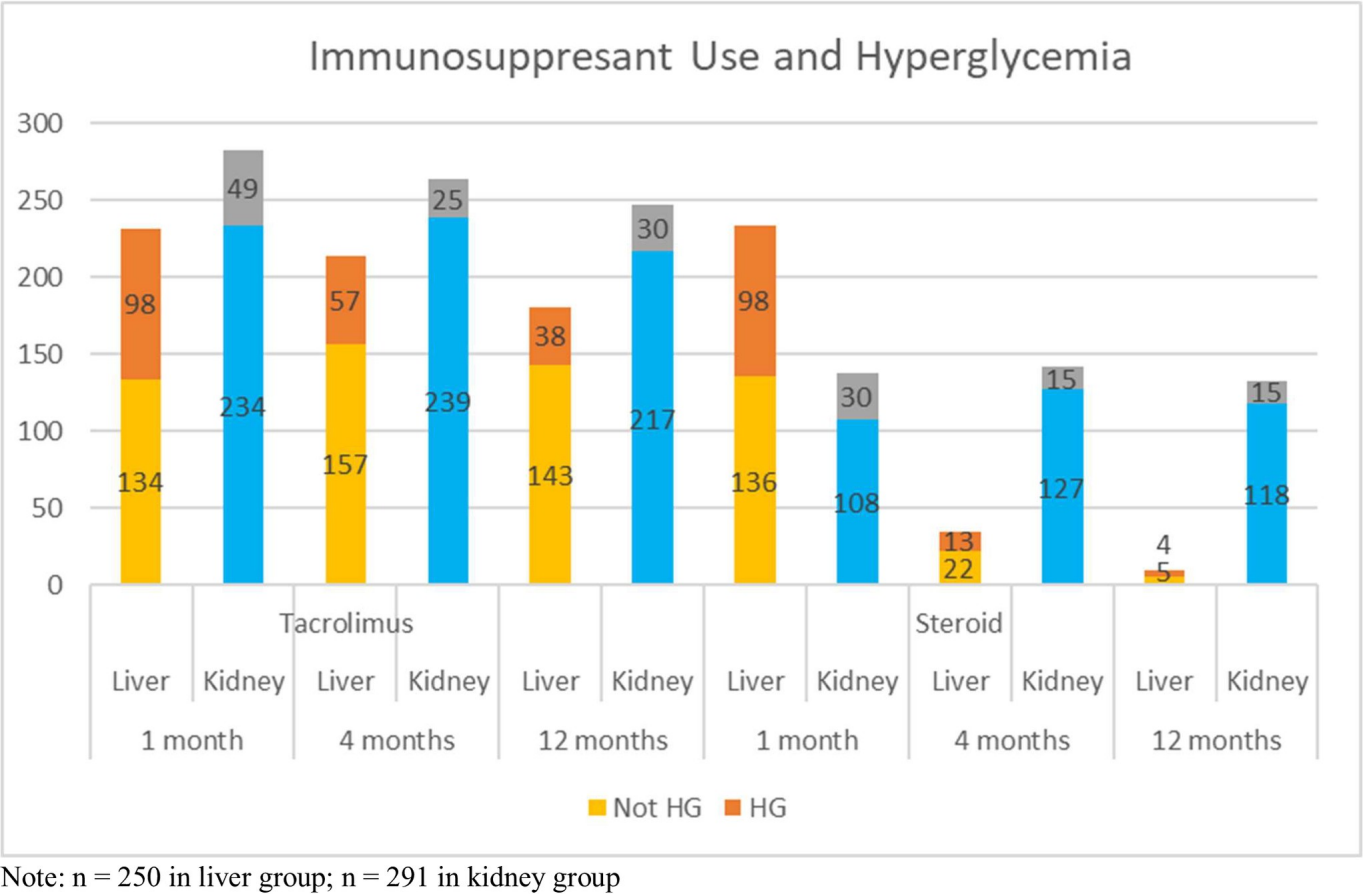
Number of liver and kidney transplantation patients who used immunosuppressive agents at 1,4, and 12 months, split by having hyperglycemia (HG) or not at each follow-up.

Fig 3 demonstrates the use of tacrolimus, steroids, and sirolimus at each follow-up, split by whether or not a patient was classified as hyperglycemic at that time. Compared to the kidney cohort, the percentage of liver transplant patients on tacrolimus decreased faster over the 3 follow-ups (92.8%, 85.6%, and 72.4% in liver vs 97%, 90.4%, and 84.6% in kidney). In addition, the percentage of patients on tacrolimus with hyperglycemia was consistently higher in the liver cohort (42.4%, 26.6%, and 21% vs 17.3%, 9.5%, and 12.1%) than the kidney group.

Liver transplantation protocols called for tapering off of steroids by 4 months. While 93.6% of liver patients were on steroids at 1 month, this percentage dropped to just 14% at 4 months and 3.6% at 1 year. This contrasts steroid use in the kidney group in which steroid use was relatively stable at all 3 follow-ups (47.3%, 48.6%, and 45.5%). Among patients on steroids, the percentage with hyperglycemia decreased in the kidney cohort (21.7%, 10.6%, and 11.3%), while hyperglycemia in liver transplant steroid users increased over from 1 month to 1 year (41.9%, 37.1%, and 44.4%).

### Predictors of PTDM

Finally, an adjusted analysis was conducted (Table 3) to determine variables associated with developing PTDM by 1 year of follow-up for liver transplant patients. The variables that were significantly associated with PTDM diagnosis were age, BMI, type of organ transplanted, insulin use at 1 month, patient stay mean glucose in the hospital, and blood glucose 24 hours before discharge. For every 5-year increase in age, the odds of PTDM developing by 1-year post-transplant increased 21% (OR 1.21, 95% CI 1.08–1.35, p < .01). A unit increase in BMI increased the odds of PTDM by 5% (OR 1.05, 95% CI 1.01–1.10, p = .02). Use of insulin at 1 month posttransplant was associated with an increased odds of PTDM by 576% (OR 5.76, 95% CI 3.18–10.42, p < .01), while use of steroids, tacrolimus, and sirolimus at 1 month had no effect on PTDM odds. Inpatient glucometrics were strong predictors of eventual PTDM development. For every 10 mg/dL increase in patient stay mean glucose, the odds of developing PTDM increased by 32% (OR 1.32, 95% CI 1.15–1.51, p = < .01), and for every 10 mg/dL increase in mean glucose 24h prior to discharge, the risk increased by 15% (OR 1.15, 95% CI 1.07–1.24, p = < .01). Finally, having a liver transplant was associated with a 337% increase (OR 3.37, 95% CI 1.38–8.25, p = < .01) in odds of PTDM. Race, pre-transplant HbA1c, and gender were not predictors of PTDM development. The risk was also equivalent whether donor was living or deceased (Table 3).

**Table 3:** Logistic regression coefficients for association between patient characteristics/treatment and probability of PTDM diagnosis in first year after transplant.

Variable	Adjusted Odds Ratios	95% CI	P-value
Age, per 5 years	1.21	1.08–1.35	< .01
BMI, kg/m^2^	1.05	1.01–1.10	.02
Race (White)	.60	.33–1.10	.10
Male (vs. Female)	1.41	.85–2.33	.18
Live Donor (vs. Deceased)	0.97	.57–1.66	.92
Liver (vs. Kidney)	3.37	1.38–8.25	< .01
Pre-transplant HbA1c (pre-transplant), %	.90	.51–1.57	.71
Tacrolimus Use at 1 month (vs. no use)	3.04	.30–30.41	.34
Sirolimus Use at 1 month (vs. no use)	1.51	.13–16.83	.74
Steroid Use at 1 month (vs. no use)	1.44	.70–2.96	.32
Insulin Use at 1month (vs. no use)	5.76	3.18–10.42	< .01
Inpatient mean glucose post-transplant, per 10 mg/dL	1.32	1.15–1.51	< .01
Mean glucose 24 hours before hospital discharge, per 10 mg/dL	1.15	1.07–1.24	< .01
Transplant Year (vs. 1999)	.88	.77–1.01	.06

Note: Adjusted models control for available pre-transplant patient characteristics (age, sex, race, BMI, donor status, pre-transplant HbA1c, transplant year, and transplant type)

## Discussion

All patients undergoing solid organ transplant without a prior history of DM are at risk of developing PTDM. This risk results from a combination of traditional factors (e.g., obesity, race/ethnicity) and immunosuppressant medications known to affect glucose metabolism [27, 28]. Once PTDM develops, these patients would then be classified and managed under the same standards as anyone with diabetes. These standards include the potential need for pharmacological agents to control hyperglycemia to appropriate targets, diabetes self-management education (DSME), performance of self-monitoring of blood glucose (SMBG), and interventions to reduce risk of micro- and macrovascular complications [29]. Given the complexity of diabetes care, and that provision of DSME requires investment of manhours by specially trained practitioners (e.g. certified diabetes educators), understanding who might develop PTDM is essential for appropriate allocation of health care resources.

Most studies of PTDM in kidney and liver transplant patients have typically involved analyses of the single organ. To the authors’ knowledge, a comparative study of liver and kidney transplants with regards to PTDM has not been undertaken. The time course for development of PTDM may not be identical between the two, and insulin needs may differ over the short term (1-year) follow-up. Understanding any potential differences can shed insight into how to modify treatment and follow-up algorithms according to the transplant type. Prior analysis by Boloori at al. [22] demonstrated a variety of PTDM features in kidney transplantation patients. Using a newly available dataset from the same institution on liver transplantation patients enabled a more comparable characterization of similarities and differences across liver and kidney transplant populations.

The analyses demonstrated significant differences in PTDM risk between the two transplant populations. Liver transplant recipients had a 337% greater odds of developing PTDM compared to kidney recipients. There are multiple possible reasons for the differential risk between kidney and liver transplantations that warrant further investigation. First, steroid protocols differ for liver transplant patients compared to renal patients which may impact hyperglycemic effects. Secondly, there may be inherent differences in glucose metabolism between kidney and liver transplant recipients which are not accounted for in this analysis. Finally, lifestyle differences may exist between these populations as a result of different recovery times from transplant allowing for modifications in diet/exercise.

Different patterns of hyperglycemia were also noted. Glucose levels were highest at 1 month in the kidney transplant group, and then declined thereafter. In the liver cohort, glucose values did not change over the follow-up time period. These differences in glucose between the two transplant cohorts may well have been due to their respective immunosuppression protocols. We also demonstrated large differences in the time course and remitting/relapsing nature of hyperglycemia between kidney and liver transplant patients. This analysis illustrates further the complex nature of post-transplant hyperglycemic and the need to better understand how the diabetogenic effects of immunosuppressive drugs impact short terms changes in glycemic control.

In the adjusted analysis, insulin use at 1 month was predictive of PTDM risk. The proportion of patients on insulin differed between the kidney and liver transplant patients. Among patients who did not develop PTDM, both renal and liver cohorts were able to discontinue insulin after 1 month post-transplant. A similar percentage of renal transplant patients who did have PTDM remained on insulin therapy across the three follow-up periods, while the proportioned declined in liver patients. The reason underlying these different proportions of insulin use cannot be ascertained from this analysis. In addition to HbA1c and blood glucose values, insulin therapy in the outpatient setting is driven by results and patterns of SMBG, which are not available here. It should be noted, however, that both groups had around 30% of recipients on insulin therapy at 1-year, meaning a majority of recipients had their PTDM adequately managed without antidiabetic agents, potentially through diet and exercise.

In 2014, the International Consensus Meeting on Post-transplantation Diabetes Mellitus published recommendations and future direction on dealing with PTDM in the medical community [5]. Among their suggestions was potential expansion of screening for PTDM using strategies such as glucose monitoring, and HbA1c measurement in the early post-transplant period. In addition, oral glucose tolerance tests (OGTT) represent another option to potentially prevent misdiagnosis prior to transplant, although they are more cumbersome to perform and are not routinely done in the clinical setting. Moreover, given the time span that can occur between an initial transplant evaluation and day of transplant, the original OGTT may no longer reflect the current glucose tolerance status. Inpatient glucometrics have typically not been included when examining the risk of PTDM. The inpatient setting immediately following a transplant represents an early opportunity to identify those at risk for PTDM so that DSME can be implemented. DSME encompasses skills such as how to conduct SMBG, how to recognize hypoglycemia, and insulin administration technique. Both the patient stay mean glucose and the mean glucose late in the hospital stay (24 hours prior to discharge) were significantly associated with the odds of developing PTDM in this analysis. Moreover, as cited previously, the majority of kidney and liver transplant patients without a prior history of DM are discharged on insulin. Together, this data affirms the need to invest hospital resources to assure that DSME is conducted prior to discharge. Controlling inpatient hyperglycemia could be a modifiable risk factor, however, whether doing so reduces PTDM risk is not known.

There are limitations of this study that should be noted. First, the sample size of patients who developed PTDM may be underpowered to detect some of the significant differences in outcomes in the time points where no significant difference was detected. Previous analysis by Boloori et al. [22], which analyzed kidney data only, utilized an imputation method to account for missing values and increase sample size. However, we chose to use raw data for both kidney and liver analyses to ensure validity of direct comparison. In some cases, the differences that were detected as statistically significant may not be clinically meaningful. Second, these data were collected from a single medical institution in the United States and may not be representative of hospitals in the United States as a whole. Thus, findings should not be generalized at this time. The analysis also uses retrospective data which is not always fully collected with specific analyses in mind. For example, indications for transplantation and known risk factors for DM such as presence of the hepatitis C virus (HCV) or alcoholism were not available for use in this study. Further studies should look at how changing trends in transplantation indication may impact these comparisons. On the other hand, many typical risk factor variables found in PTDM literature are included. Data collection differences between organs did not allow for exact comparisons between the two organs. As a result, we rely on assumptions such as the use of insulin therapy as a proxy for a PTDM diagnosis. Using the findings from this analysis to see how well the identified characteristics predict PTDM prospectively could be a possible next step.

These results provide the first evidence to support differential guideline-making and management between kidney and liver transplant patients to reflect differences in both the risk factors and timeline at which patients may be at risk to develop PTDM. Furthermore, these findings highlight the need for further investigation of PTDM features and outcomes across other transplanted organs, as well as novel strategies to manage PTDM risk. In addition, potential effectiveness of screening strategies to better diagnose and monitor glucose intolerance in this population should be explored. It is likely that hospitals across the United States would benefit from standardization of protocols that optimize PTDM risk management in this patient population to minimize likelihood of poor diabetes outcomes while maximizing the health of the transplanted organ.

## References

[1] Kasiske BL, Snyder JJ, Gilbertson D, Matas AJ. Diabetes mellitus after kidney transplantation in the United States. Am J Transplant. 2003;3(2):178–85. 10.1034/j.1600-6143.2003.00010.x 12603213

[2] Kasiske BL, Chakkera HA, Roel J. Explained and unexplained ischemic heart disease risk after renal transplantation. J Am Soc Nephrol. 2000;11(9):1735–43. 10966499 10.1681/ASN.V1191735

[3] Ghisdal L, Van Laecke S, Abramowicz MJ, Vanholder R, Abramowicz D. New-onset diabetes after renal transplantation: risk assessment and management. Diabetes Care. 2012;35(1):181–8. 10.2337/dc11-1230 22187441 PMC3241330

[4] Davidson J, Wilkinson A, Dantal J, Dotta F, Haller H, Hernandez D, et al New-onset diabetes after transplantation: 2003 International consensus guidelines. Proceedings of an international expert panel meeting. Barcelona, Spain, 19 February 2003. Transplantation. 2003;75(10 Suppl):SS3–24. 12775942 10.1097/01.TP.0000069952.49242.3E

[5] Sharif A, Hecking M, de Vries AP, Porrini E, Hornum M, Rasoul-Rockenschaub S, et al Proceedings from an international consensus meeting on posttransplantation diabetes mellitus: recommendations and future directions. Am J Transplant. 2014;14(9):1992–2000. 10.1111/ajt.12850 25307034 PMC4374739

[6] Baid S, Cosimi AB, Farrell ML, Schoenfeld DA, Feng S, Chung RT, et al Posttransplant diabetes mellitus in liver transplant recipients: risk factors, temporal relationship with hepatitis C virus allograft hepatitis, and impact on mortality. Transplantation. 2001;72(6):1066–72. 10.1097/00007890-200109270-00015 11579302

[7] Knobler H, Stagnaro-Green A, Wallenstein S, Schwartz M, Roman SH. Higher incidence of diabetes in liver transplant recipients with hepatitis C. J Clin Gastroenterol. 1998;26(1):30–3. 10.1097/00004836-199801000-00009 9492860

[8] Ye X, Kuo HT, Sampaio MS, Jiang Y, Bunnapradist S. Risk factors for development of new-onset diabetes mellitus after transplant in adult lung transplant recipients. Clin Transplant. 2011;25(6):885–91. 10.1111/j.1399-0012.2010.01383.x 21175848

[9] Pham PT, Pham PM, Pham SV, Pham PA, Pham PC. New onset diabetes after transplantation (NODAT): an overview. Diabetes Metab Syndr Obes. 2011;4:175–86. 10.2147/DMSO.S19027 21760734 PMC3131798

[10] Kim WR, Lake JR, Smith JM, Skeans MA, Schladt DP, Edwards EB, et al OPTN/SRTR 2015 Annual Data Report: Liver. Am J Transplant. 2017;17 Suppl 1:174–251. 28052604 10.1111/ajt.14126

[11] Hart A, Smith JM, Skeans MA, Gustafson SK, Stewart DE, Cherikh WS, et al OPTN/SRTR 2015 Annual Data Report: Kidney. Am J Transplant. 2017;17 Suppl 1:21–116. 28052609 10.1111/ajt.14124 PMC5527691

[12] Crutchlow MF, Bloom RD. Transplant-associated hyperglycemia: a new look at an old problem. Clin J Am Soc Nephrol. 2007;2(2):343–55. 10.2215/CJN.03671106 17699434

[13] Carter SA, Kitching AR, Johnstone LM. Four pediatric patients with autosomal recessive polycystic kidney disease developed new-onset diabetes after renal transplantation. Pediatr Transplant. 2014;18(7):698–705. 10.1111/petr.12332 25118046

[14] Gaynor JJ, Ciancio G, Guerra G, Sageshima J, Hanson L, Roth D, et al Multivariable risk of developing new onset diabetes after transplant-results from a single-center study of 481 adult, primary kidney transplant recipients. Clin Transplant. 2015;29(4):301–10. 10.1111/ctr.12510 25581205

[15] Kuo HT, Lau C, Sampaio MS, Bunnapradist S. Pretransplant risk factors for new-onset diabetes mellitus after transplant in pediatric liver transplant recipients. Liver Transpl. 2010;16(11):1249–56. 10.1002/lt.22139 21031540

[16] Luan FL, Langewisch E, Ojo A. Metabolic syndrome and new onset diabetes after transplantation in kidney transplant recipients. Clin Transplant. 2010;24(6):778–83. 10.1111/j.1399-0012.2009.01194.x 20047609 PMC3831507

[17] Lv C, Chen M, Xu M, Xu G, Zhang Y, He S, et al Influencing factors of new-onset diabetes after a renal transplant and their effects on complications and survival rate. PLoS One. 2014;9(6):e99406 10.1371/journal.pone.0099406 24911157 PMC4050028

[18] Palepu S, Prasad GV. New-onset diabetes mellitus after kidney transplantation: Current status and future directions. World J Diabetes. 2015;6(3):445–55. 10.4239/wjd.v6.i3.445 25897355 PMC4398901

[19] Park SC, Yoon YD, Jung HY, Kim KH, Choi JY, Park SH, et al Effect of transient post-transplantation hyperglycemia on the development of diabetes mellitus and transplantation outcomes in kidney transplant recipients. Transplant Proc. 2015;47(3):666–71. 10.1016/j.transproceed.2014.11.053 25891707

[20] Rodrigo E, Fernandez-Fresnedo G, Valero R, Ruiz JC, Pinera C, Palomar R, et al New-onset diabetes after kidney transplantation: risk factors. J Am Soc Nephrol. 2006;17(12 Suppl 3):S291–5. 17130277 10.1681/ASN.2006080929

[21] Pirsch JD, Henning AK, First MR, Fitzsimmons W, Gaber AO, Reisfield R, et al New-Onset Diabetes After Transplantation: Results From a Double-Blind Early Corticosteroid Withdrawal Trial. Am J Transplant. 2015;15(7):1982–90. 10.1111/ajt.13247 25881802

[22] Boloori A, Saghafian S, Chakkera HA, Cook CB. Characterization of Remitting and Relapsing Hyperglycemia in Post-Renal-Transplant Recipients. PLoS One. 2015;10(11):e0142363 10.1371/journal.pone.0142363 26551468 PMC4638338

[23] Boloori A, Saghafian S, Chakkera HA, Cook CB. Data-Driven Management of Post-Transplant Medications: An Ambiguous Partially Observable Markov Decision Process Approach. Manufacturing and Service Operations Management (forthcoming). 2019.

[24] Chakkera HA, Weil EJ, Castro J, Heilman RL, Reddy KS, Mazur MJ, et al Hyperglycemia during the immediate period after kidney transplantation. Clin J Am Soc Nephrol. 2009;4(4):853–9. 10.2215/CJN.05471008 19339426 PMC2666437

[25] Werner KT, Mackey PA, Castro JC, Carey EJ, Chakkera HA, Cook CB. Hyperglycemia during the immediate period following liver transplantation. Future Sci OA. 2016;2(1):FSO97 10.4155/fsoa-2015-0010 28031946 PMC5138006

[26] Cook CB, Castro JC, Schmidt RE, Gauthier SM, Whitaker MD, Roust LR, et al Diabetes care in hospitalized noncritically ill patients: More evidence for clinical inertia and negative therapeutic momentum. J Hosp Med. 2007;2(4):203–11. 10.1002/jhm.188 17683100

[27] Subramanian S, Trence DL. Immunosuppressive agents: effects on glucose and lipid metabolism. Endocrinol Metab Clin North Am. 2007;36(4):891–905; vii. 10.1016/j.ecl.2007.07.003 17983927

[28] Penfornis A, Kury-Paulin S. Immunosuppressive drug-induced diabetes. Diabetes Metab. 2006;32(5 Pt 2):539–46. 17130815 10.1016/s1262-3636(06)72809-9

[29] American Diabetes A. Standards of Medical Care in Diabetes-2017 Abridged for Primary Care Providers. Clin Diabetes. 2017;35(1):5–26. 10.2337/cd16-0067 28144042 PMC5241768

